# Supplementary figures and images for: Enhanced M2 Polarization of Retinal Microglia in Streptozotocin-Induced Diabetic Mice upon Autoimmune Stimulation
Source: Biomedicines. 2025 Aug 22;13(9):2049. doi: 10.3390/biomedicines13092049 (PMC12467324; doi:10.3390/biomedicines13092049)

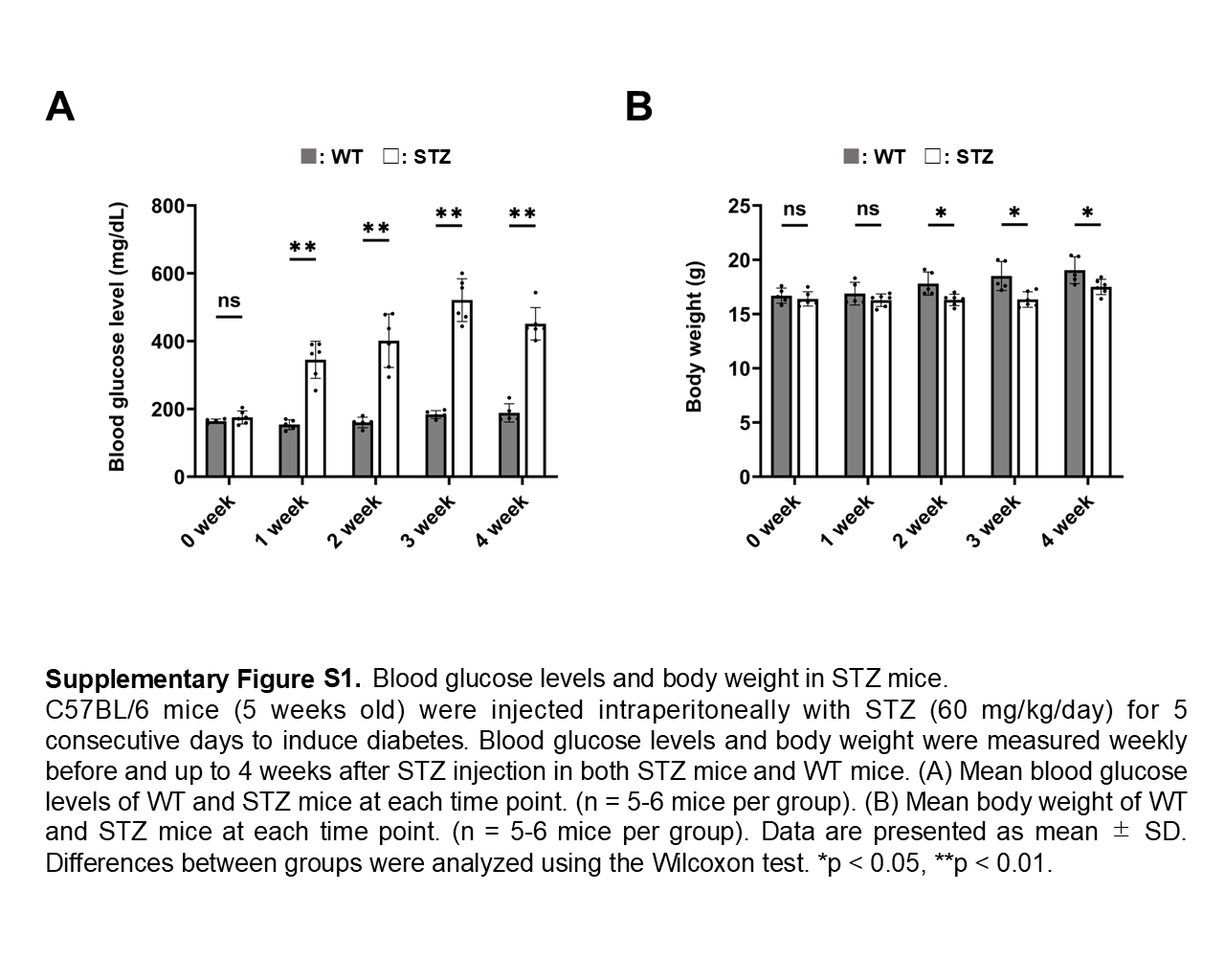

Supplement: Supplementary file 1 [file biomedicines-13-02049-s001.zip › Figure S1.TIF]

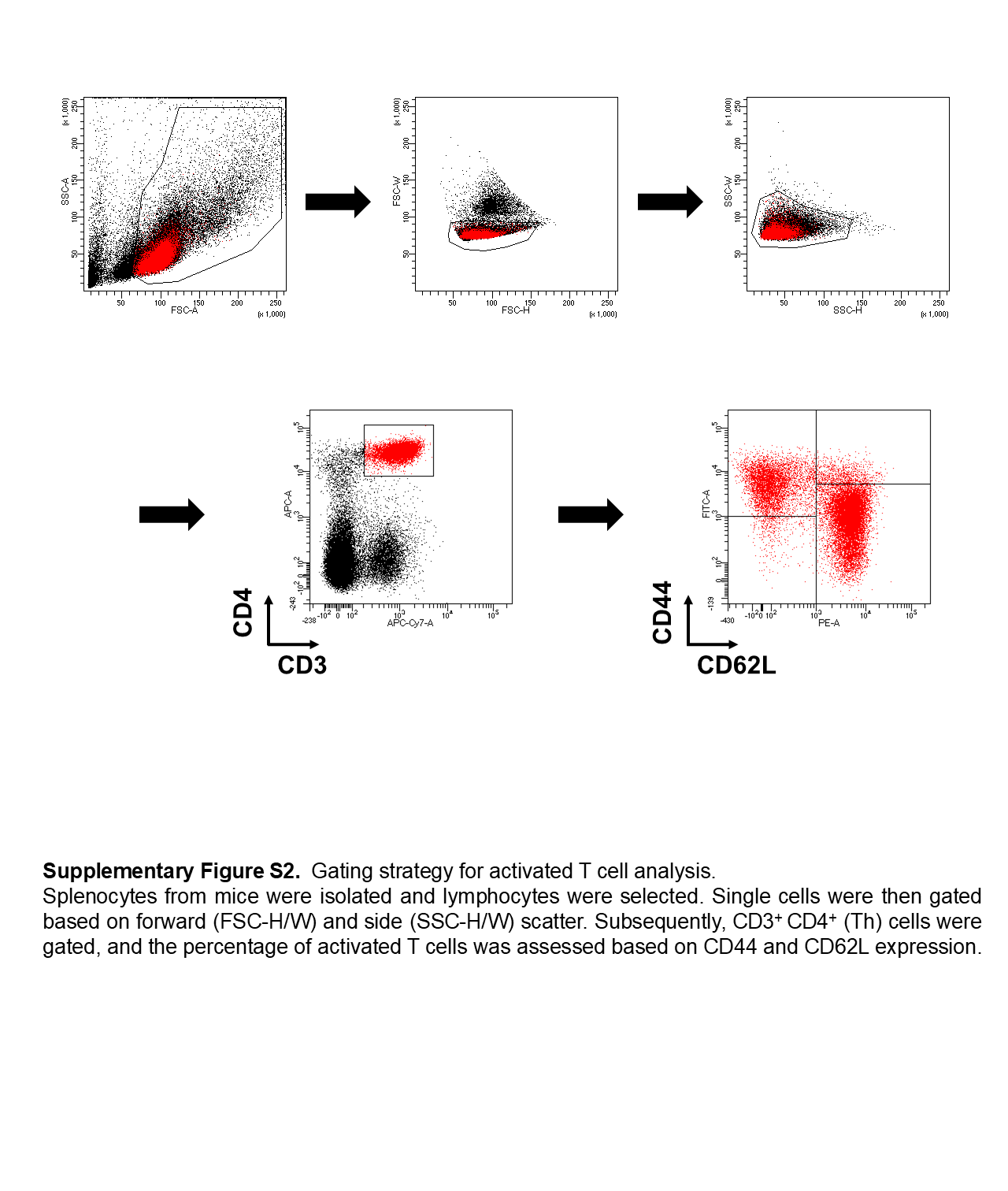

Supplement: Supplementary file 1 [file biomedicines-13-02049-s001.zip › Figure S2.TIF]

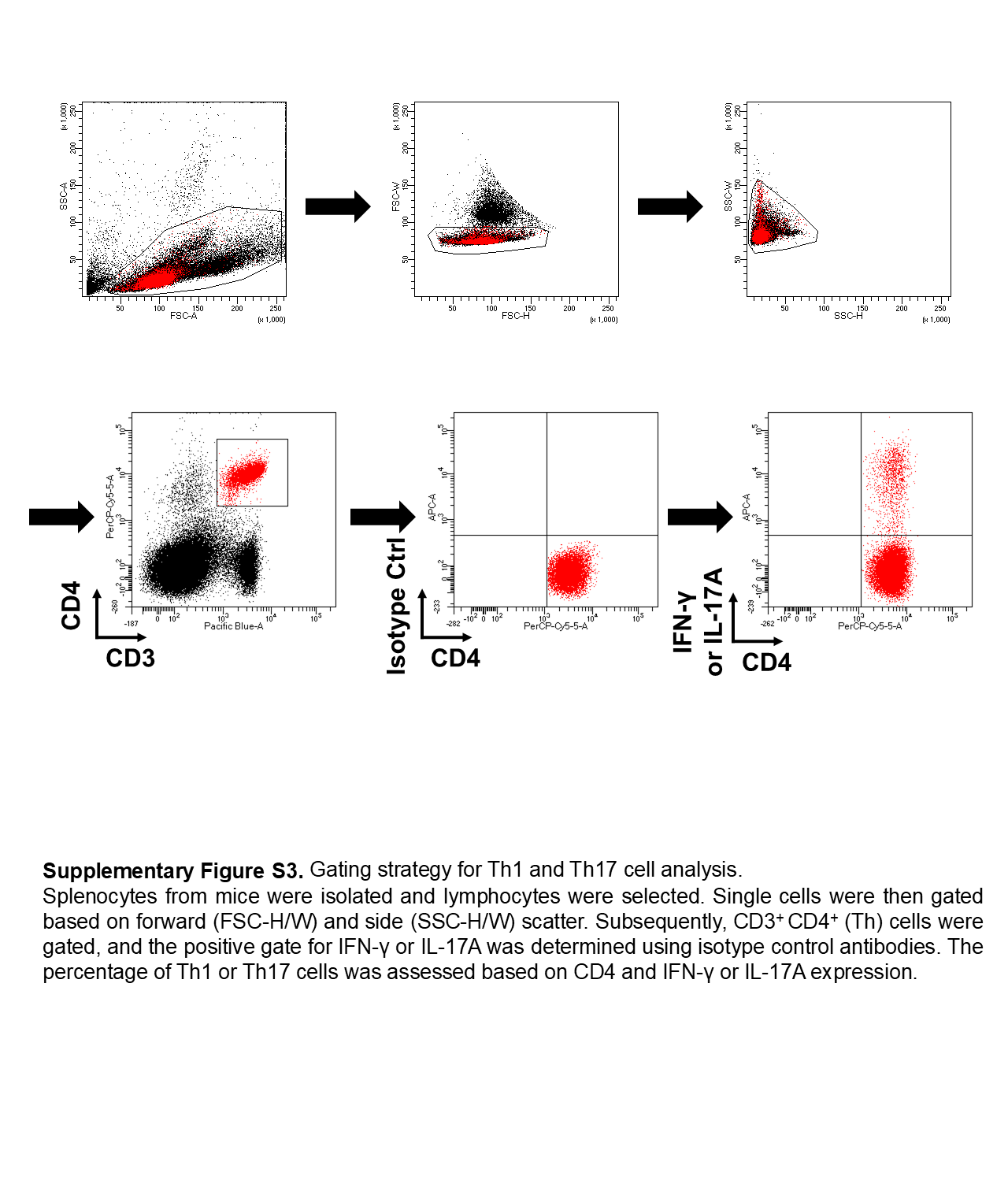

Supplement: Supplementary file 1 [file biomedicines-13-02049-s001.zip › Figure S3.TIF]

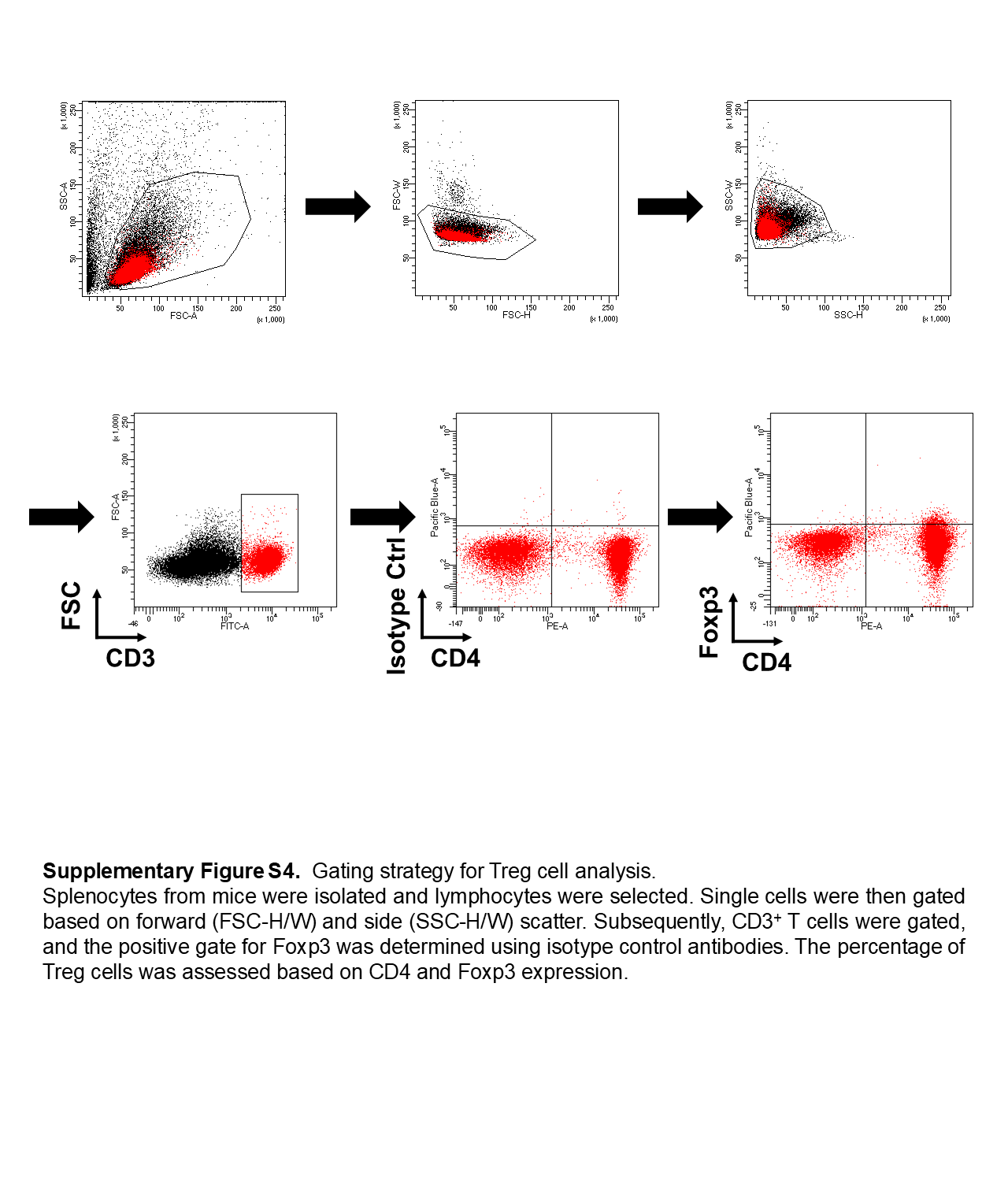

Supplement: Supplementary file 1 [file biomedicines-13-02049-s001.zip › Figure S4.TIF]

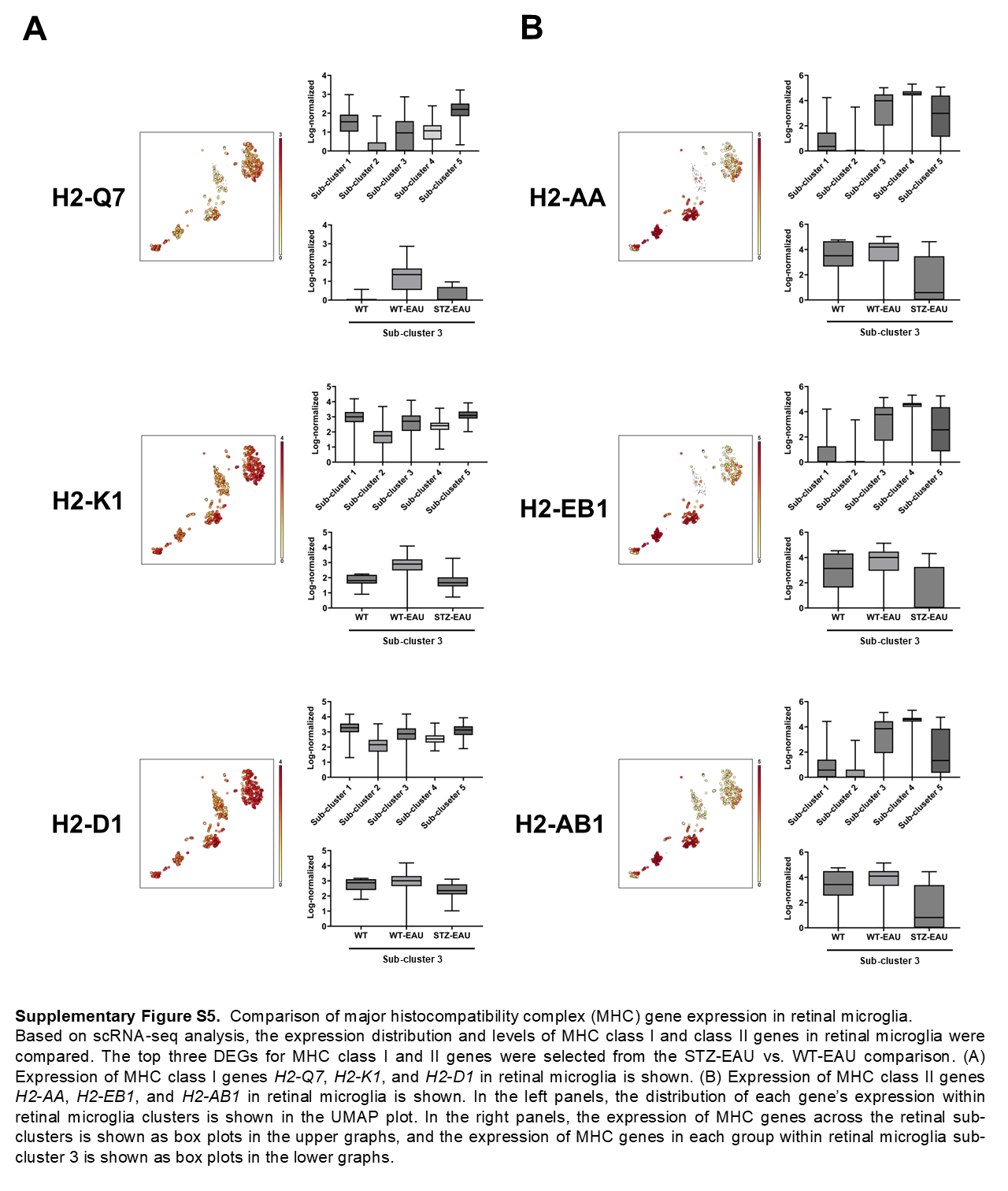

Supplement: Supplementary file 1 [file biomedicines-13-02049-s001.zip › Figure S5.TIF]
